# Phosphate ion removal from aqueous solution using snail shell dust: biosorption potential of waste shells of edible snails

**DOI:** 10.1039/d2ra03852h

**Published:** 2022-10-25

**Authors:** Pranesh Paul, Suprio Parbat, Gautam Aditya

**Affiliations:** Department of Zoology, University of Calcutta 35, Ballygunge Circular Road Kolkata – 700019 India plpranesh@gmail.com suprioparbat29@gmail.com gautamaditya2001@gmail.com +91 3324614849 +91 3324615445 extn 284

## Abstract

The freshwater snails, *Filopaludina bengalensis* and *Pila globosa* are widely used for human consumption and as a feed in aquaculture in India and Bangladesh. The generation of shells as a waste product following meat extraction from the live snails incites their utilisation as a potential biomaterial. Shell dust was prepared from the dried shells of *F. bengalensis* (FSD) and *P. globosa* (PSD) and employed for phosphate adsorption from aqueous solutions. Batch adsorption experiments were performed to examine the effects of various experimental conditions, such as biosorbent dose, agitation speed, temperature, contact time, pH, initial concentration of phosphate ions, and presence of co-existing ions. SEM, EDS, ICP-OES, FTIR, and XRD results indicated that phosphate ions were adsorbed onto the surface of shell dust particles. The experimental data fitted with the Langmuir isotherm with a maximum adsorption capacity of 62.50 and 66.66 mg g^−1^ for FSD and PSD. The pseudo-second order kinetic model was well fitted, indicating the chemical adsorption process, and the thermodynamic parameters indicated that the adsorption mechanism of phosphate was spontaneous, feasible, and endothermic. Therefore, the results have established the potentiality of the waste shells of edible snails to be used as an eco-friendly and low-cost biosorbent for phosphate removal from wastewater.

## Abstract 2

The waste shells of *Filopaludina bengalensis* and *Pila globosa* can be used as an eco-friendly and low-cost biosorbent for phosphate removal from eutrophicated water or industrial wastewater.

## Graphical abstract

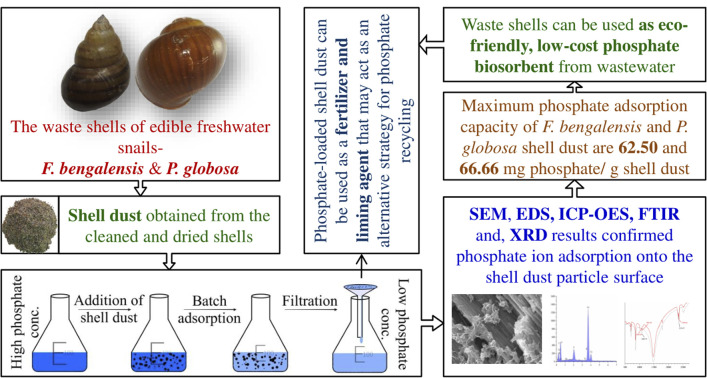

## 1. Introduction

Phosphorus is an essential nutrient for the growth of organisms and the sustenance of ecosystems. A moderate amount of phosphorus, along with other macro- and micronutrients, is crucial for the continual growth and productivity of aquatic ecosystems. More than 99% of naturally occurring phosphorous is present in inorganic phosphates or as organic phosphate ester forms, and organic forms of phosphate are more mobile than the inorganic forms in the environment.^1^ The free phosphate ions have a high negative charge density, which restricts the mobility of phosphate in the environment as it can bind to positively charged surfaces. In the presence of fewer absorbing surfaces and the constant addition of phosphate in the waterbodies, organisms can obtain phosphate effortlessly, even in lower phosphate concentrations which turn aquatic ecosystems extremely sensitive to phosphate contamination and eutrophication.^1^ Hence, when in excess amount (above 0.02 mg L^−1^), phosphorus is considered a pollutant as the unwarranted phosphorus can lead to eutrophic water bodies.^2,3^ Among several factors, excessive plant production, increased anoxic events, harmful algal bloom, and depletion of the dissolved oxygen are the consequence of eutrophication, which have multiple harmful impacts on the aquatic organism.^4,5^ From the anthropogenic perspective, eutrophication results in economic losses in aquaculture, wildlife production and includes the cost of water purification for human and industrial use.

Worldwide, an enormous amount of phosphate-containing industrial and domestic wastewater and agricultural runoffs get discharged into natural water bodies, including lakes and rivers.^6–8^ Considering the harmful impacts of excessive phosphate in the water bodies, the Environmental Protection Agency (EPA) declared the maximum permissible level and discharge limit to be 0.1 mg L^−1^ and less than 0.05 mg L^−1^ phosphate ions, respectively. Hence, the development of certain proficient methods for removing phosphate from the water and wastewaters is essential to sustain aquatic ecosystems. In the last few decades, various methods, such as chemical precipitation, flocculation, membrane purification, nanofiltration, electrodialysis, and reverse osmosis, have been used to remediate phosphate from industrial wastewater.^9,10^ However, those conventional techniques have their own limitations. For instance, the chemical precipitation produces an additional amount of sludge, membrane separation is expensive and requires high initial investments, and few techniques produce secondary pollutants, affect the pH of the effluent and require the addition of chemicals before discharge.^9,11^ On the other side, bioremediation is an alternative, relatively low-cost option over the conventional techniques that offer the possibility to destroy or render several contaminants and generally have a high public acceptance.

Although algae,^12,13^ bacteria,^14,15^ and plants^16,17^ were widely used for bioremediation, consideration of animal models is rare due to ethical concerns, and a majority of such animals are cultured and harvested for human consumption.^18^ However, the animal waste materials from households, restaurants, and industries comprise calcium carbonate, chitin, and fibrous protein, which can be used as a potential biomaterial for pollutant removal. For example, mollusc shells, crab shells, fish scales, poultry bird feathers, and eggshells have been used as adsorbents of various pollutants.^19–22^ As the high costs of commercial adsorbents limit their application in developing countries,^23^ agricultural/food wastes/by-products provide an emerging trend for their use as biosorbent to diminish the cost of treatment.^24–26^

Numerous freshwater snail species from wetlands, ponds, rivers, and rice fields are harvested in Asian countries, including India, for consumption.^27–30^ The food quality of many freshwater snail species enables us to consider the snails as a cheap source of minerals and protein. The flesh derived from freshwater snails like *Filopaludina bengalensis* (Lamarck, 1822) (Gastropoda: Viviparidae) and *Pila globosa* (Swainson, 1828) (Gastropoda: Ampullaridae) is widely used for human consumption and as a feed in the farming of freshwater prawns, fish and ducks in India and Bangladesh.^31,32^ In the south-western districts of Bangladesh, around 9750 kg of snail meat per hectare per year is being provided to the juvenile prawns.^31^ The generation of shells as a waste product following the extraction of meat from the live edible snails incites their utilisation to make sustainable utility of the natural resources. The discarded shell of the snails has been recognised and used as biological material in multiple ways. For instance, the snail shells were used as a biocatalyst for biodiesel production,^33^ PVC composite material,^34^ the hydroxyapatite crystals obtained from shells were proposed as prospective orthopedic applications,^35^ and the efficacy of shell dust as biosorbent of heavy metal ions was also studied considerably.^22,36–39^ In a recent study, the structural and mechanical properties of the shells of *F. bengalensis* and *P. globosa* were revealed,^40^ which supported their potential multifunctional roles, including heavy metal removal, biocatalyst for energy production and biomedical usages.^33^ Therefore, the shells of *F. bengalensis* and *P. globosa* were chosen and employed for phosphate adsorption from the aqueous solution, and their potentiality was examined using the batch adsorption experiments by fitting the experimental data with various adsorption isotherms, kinetic models, and thermodynamic parameters.

## 2. Materials and methods

### 2.1. Preparation of shell dust

**Fig. 1:**
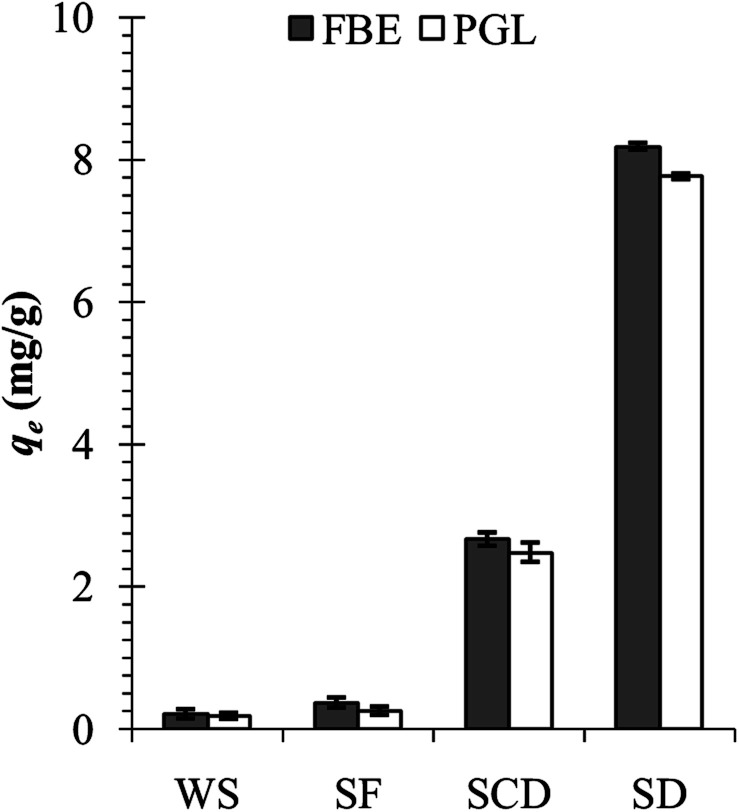
Phosphate adsorption capacity of the whole shell (WS), shell flakes (SF), coarse shell dust (SCD), and fine shell dust (SD) of *F. bengalensis* (FBE) and *P. globosa* (PGL).

The waste shells of edible freshwater snails, *F. bengalensis* (shell length – 26.9 ± 0.6 (mean ± SE) mm, shell weight – 1.3 ± 0.1 g), and *P. globosa* (shell length – 49.2 ± 0.8 mm, shell weight – 8.8 ± 0.4 g) were collected from the fish markets of Howrah, West Bengal, India. In the laboratory, the shells were initially cleaned with running tap water to remove any dirt or remaining tissues and later with distilled water. After cleaning, the shells were dried in a hot air oven (40 °C) for the next two days. The completely dried shells were pulverised to shell flakes (5–10 mm), coarse shell dust (1 mm to >200 μm), and fine shell dust (<200 μm) by using mortar and pestle and sieved through different pore sizes of strainers. Initially, the fine shell dust of *F. bengalensis* (henceforth FSD) and *P. globosa* (henceforth PSD) showed higher phosphate adsorption capacity than the coarse shell dust, shell flakes, and whole shells (Fig. 1), therefore, the rest of the experiment was conducted with fine shell dust only.

### 2.2. Preparation of phosphate solution

All chemicals used in the experiment were of analytical grade and brought from Sigma-Aldrich Chemicals Private Limited. The phosphate stock solution was made by dissolving an appropriate amount of monopotassium phosphate (KH_2_PO_4_) in 1 litre of double-distilled water. The stock solution was further diluted to working phosphate solutions (100–1000 mg L^−1^) using double distilled water as and when required. The dominance of phosphate species varies with the pH of the solution, *i.e.*, H_2_PO^−^_4_ dominates at pH < 7, HPO^2−^_4_ dominates at 7 < pH < 10 and PO^3−^_4_ dominates at pH > 12.5.^41,42^

### 2.3. Characterisation of shell dust

The fine shell dust, *i.e.*, FSD and PSD (before and after phosphate adsorption), were platinum-coated, and micrographs were taken by scanning electron microscope (SEM) (EVO 18 special edition, Zeiss) to observe the surface structure and morphology. The elemental composition of FSD and PSD (before and after phosphate adsorption) was determined by energy-dispersive X-ray spectroscopy (EDS) (SmartEDX, Zeiss).

The elemental content (Na, K, Mg, Mn, and P) of FSD and PSD was further quantified using an Inductively coupled plasma-optical emission spectrometry (ICP-OES) (Avio 200, PerkinElmer). 20 mg shell dust (before and after phosphate adsorption) was acid digested using 5 mL of HCl and HNO_3_ (2 : 1 by volume). 1 mL H_2_O_2_ was added following the heating on 100 °C for 2 minutes, and the solution was further heated for another 1 minute. The completely digested samples were cooled at room temperature, transferred to volumetric tubes, and diluted to 20 ml with demineralised water. The samples were introduced to the ICP-OES using a peristaltic pump-connected autosampler at a flow rate of 1 mL min^−1^ into the argon plasma through a cross-flow nebuliser and spray chamber. The instrument operating conditions were recommended by the manufacturer, which were set as plasma power of 1.5 kW, plasma gas flow of 8 L min^−1^, nebuliser gas flow of 0.7 L min^−1^, and auxiliary gas flow of 0.2 L min^−1^. All elements were detected at specific wavelengths (Na – 589.592 nm, K – 766.490 nm, Mg – 285.213 nm, Mn – 257.610 nm, and P – 213.617 nm) to gain maximum signal intensity and minimum spectral overlap, and the emission line background corrections were performed manually. The calibration curves for each element were prepared by diluting a standard solution (Periodic table mix 1 for ICP, TraceCERT®, Sigma-Aldrich) to different concentrations (in a range of 0.1 to 10 mg L^−1^).

99% KBr powder and 1% FSD and PSD (by weight) were thoroughly mixed, and KBr pellets (13 mm in diameter) were made using a standard hydraulic press device under a pressure of 100 kN cm^−2^ for Fourier transform infrared spectrum analysis.^40^ Fourier transform infrared (FTIR) spectra of FSD and PSD (before and after phosphate adsorption) were collected in the range of 500–3000 cm^−1^ at 1 cm^−1^ resolution on a Jasco FT/IR-6300 FTIR spectrometer.

The crystalline phase compositions of FSD and PSD (before and after phosphate adsorption) were determined by powder X-ray diffraction (XRD) analysis (using Smartlab XRD, Rigaku). Phase composition data acquisition was carried out by stepwise scanning mode, in steps of 0.02° scattering angles (2*θ*) at the speed of 3° min^−1^, ranging from 10° to 90° by using monochromatic radiation of Cu-Kα (*λ* = 1.5406 Å) at 40 kV and 20 mA. The phase identification of the X-ray diffractogram was performed using Match! software.^43^

### 2.4. Batch adsorption experiment and analytical methods

The batch adsorption experiment was performed in 250 mL Erlenmeyer flasks containing 100 mL phosphate solution. The flasks were sealed tightly with aluminium foil and agitated in a shaking incubator. The effects of different biosorbent doses, agitation speed, pH of phosphate solution, temperature, initial concentration of phosphate solution, contact time, and presence of other coexisting ions on the phosphate adsorption potential of FSD and PSD were evaluated. After agitation with fixed parameters, the solution was filtered using Whatman® filter paper, and the phosphate concentration in the solution was determined following the method of Murphy and Riley.^44^ In the solution, phosphate ions react with ammonium molybdate in the presence of ascorbic acid and antimony and yield a blue-purple colour.^44^ The absorbance of each solution was measured with a UV-visible spectrophotometer (Labman, India) at 880 nm wavelength.^44^ The phosphate concentration was calculated using a calibration curve prepared by plotting the absorbance of the solution measured at different concentrations of phosphate against each other (*R*^2^ = 0.990). The adsorption capacity of the shell dust (*q*_e_ mg g^−1^) (eqn (1)) and percent phosphate removal from the phosphate solution (eqn (2)) were calculated using1
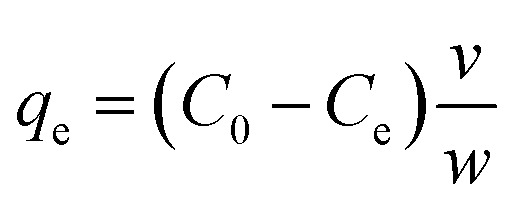
2
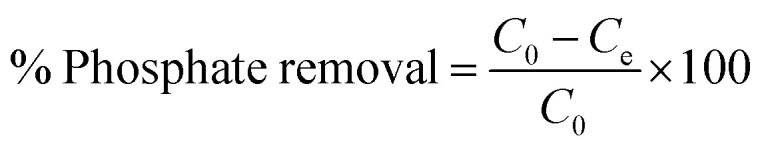
where, *C*_0_ = initial concentration of phosphate ions (mg L^−1^), *C*_e_ = final concentration of phosphate ions (mg L^−1^), *v* = volume (in L) of the phosphate solution, and *w* = weight (in g) of the biosorbent provided in the batch adsorption experiment.^23–26,36–39^

#### 2.4.1. Effect of biosorbent dose

The batch adsorption experiment was performed with different amounts of shell dust (50, 100, 250, 500, 750, and 1000 mg per 100 mL phosphate solution) to determine the effect of biosorbent doses. The agitation speed (150 rpm), pH (6), temperature (30 °C), initial concentration of phosphate ion (100 mg L^−1^), and contact time (60 minutes) remained constant in all instances.

#### 2.4.2. Effect of agitation speed

The effect of the agitation speed on the phosphate biosorption onto shell dust was evaluated by varying agitation speed (50, 100, 150, and 200 rpm) while maintaining the other parameters (biosorbent dose – 1000 mg, pH – 6, temperature – 30 °C, initial concentration of phosphate ion – 100 mg L^−1^ and contact time – 60 minutes).

#### 2.4.3. Effect of pH of phosphate solution

The effect of pH (2, 4, 6, and 8) on shell dust mediated phosphate adsorption was investigated with fixed parameters, *i.e.*, amount of shell dust (1000 mg), agitation speed (150 rpm), temperature (30 °C), initial concentration of phosphate ion (100 mg L^−1^) and contact time (60 minutes). The pH of the solutions was adjusted to the required levels using 0.1(N) HNO_3_ and 0.1(N) NaOH.

#### 2.4.4. Effect of temperature

The batch adsorption experiment was performed under different temperatures (20, 25, 30, and 35 °C) to determine the effect of temperature on phosphate adsorption. The biosorbent dose (1000 mg), agitation speed (150 rpm), pH (6), initial concentration of phosphate ion (100 mg L^−1^), and contact time (60 minutes) remained constant in all instances.

#### 2.4.5. Effect of initial concentration of phosphate solution

The effect of the initial concentration of phosphate solution (100–1200 mg L^−1^) on the phosphate biosorption onto shell dust was evaluated while maintaining the other parameters (agitation speed – 150 rpm, biosorbent dose – 1000 mg, pH – 6, temperature – 30 °C, and contact time – 60 minutes).

#### 2.4.6. Effect of contact time

The batch adsorption experiment was performed with different contact times (40, 60, 80, 100, 120, 140, 160, 180, and 200 minutes) to determine the effect of contact time on the phosphate adsorption onto shell dust. The agitation speed (150 rpm), pH (6), temperature (30 °C), biosorbent dose (1000 mg), and initial concentration of phosphate ion (100 mg L^−1^) remained constant in all instances.

#### 2.4.7. Effect of coexisting ions

To investigate the effect of other interfering ions (NO^−^_2_, NO^−^_3_ and NH^+^_4_), different concentrations of coexisting ions (10, 50, and 100 mg L^−1^) were used in the experiment, while the concentration of phosphate ion (100 mg L^−1^) remained the same in all instances. NO^−^_2_, NO^−^_3_ and NH^+^_4_ solution was prepared by dissolving an appropriate amount of potassium nitrite (KNO_2_), sodium nitrate (NaNO_3_), and ammonium chloride (NH_4_Cl) in double-distilled water.

### 2.5. Adsorption isotherms, kinetic studies, and thermodynamics parameters

Two adsorption isotherms, *i.e.*, Langmuir and Freundlich (eqn (3) and (5)) isotherms, and two kinetic models, *i.e.*, pseudo-first and pseudo-second order kinetics (eqn (6) and (7)), were selected to examine the phosphate adsorption mechanism onto FSD and PSD (Table 1). Among the adsorption isotherms, the Langmuir isotherm is applicable to monolayer adsorption, and the Freundlich isotherm describes adsorption on the heterogeneous surfaces of the adsorbate–adsorbent system.^45,46^ Similarly, the pseudo-first order kinetic model describes physical adsorption, and the pseudo-second order kinetic model focuses on chemical adsorption.^46^ The thermodynamic parameters for the phosphate adsorption onto the shell dust were derived from the experimental data using Van't Hoff and Arrhenius equations (eqn (8) and (9))^10,47,48^ (Table 1).

**Table 1:** Adsorption isotherms, kinetic models, and adsorption thermodynamics used in this experiment

(a) Langmuir isotherm	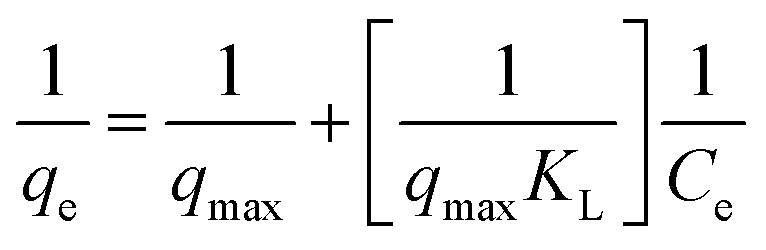 (3)
	where, *q*_e_ = adsorption capacity at equilibrium, *C*_e_ = phosphate concentration at equilibrium, *q*_max_ = maximum adsorption capacity at equilibrium, and *K*_L_ = Langmuir constant
	The favorability of this isotherm can be explained by *R*_L_ (if the value is greater than 0 but less than 1)
	*R*_L_ = 1/[1 + (1 + *K*_L_*C*_0_)] (4)
	where, *R*_L_ = equilibrium parameter and *C*_0_ = initial concentration
(b) Freundlich isotherm	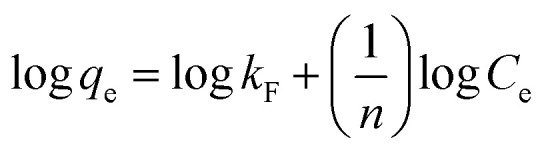 (5)
	where *k*_F_ = Freundlich constant, describing adsorption capacity of adsorbent, and *n* = adsorption intensity. 1/*n* describes the heterogeneity on which the Freundlich isotherm relies. A smaller value for 1/*n* indicates a more heterogeneous media
(c) Pseudo-first order kinetic	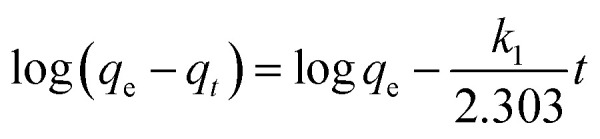 (6)
	where *q*_e_ = adsorption capacity at equilibrium (mg g^−1^), *q*_t_ = adsorption capacity at time *t* (mg g^−1^), *t* = time (minutes) and *k*_1_ = pseudo first-order rate constant
(d) Pseudo-second order kinetic	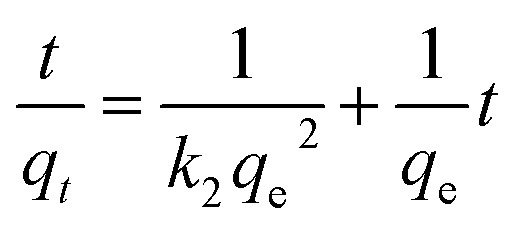 (7)
	where *q*_e_ = adsorption capacity at equilibrium (mg g^−1^), *q*_t_ = adsorption capacity at time *t* (mg g^−1^), *t* = time (minutes) and *k*_2_ = pseudo second-order rate constant
(e) Adsorption thermodynamics	Δ*G* = −*RT* ln *K*_d_ (8)
	where Δ*G* = change in Gibbs free energy, *R* = universal gas constant (8.314 J mol^−1^ K^−1^), *T* = temperature (°K), and *K*_d_ = thermodynamic equilibrium constant (*q*_e_/*C*_e_).
	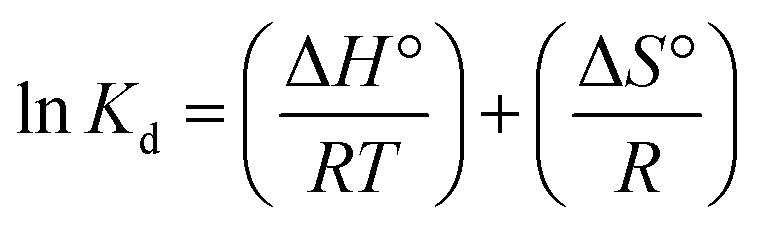 (9)
	where Δ*H*° = enthalpy of the system (how much energy is released or absorbed) and Δ*S*° = entropy (measure of randomness in the system)

## 3. Results and discussion

### 3.1. Shell dust characterisation

**Fig. 2:**
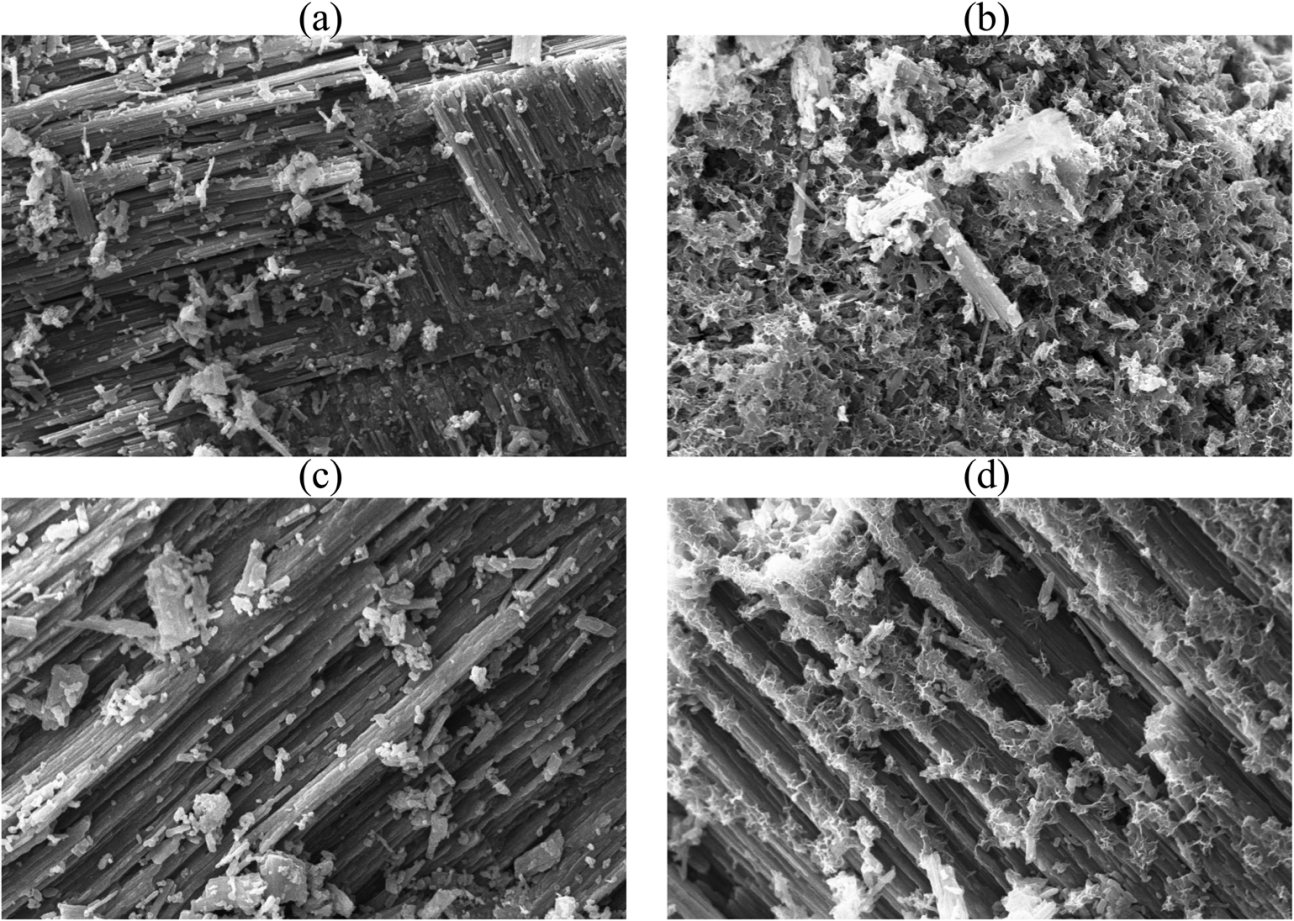
SEM micrograph of shell dust particles before ((a) FSD and (c) PSD) and after ((b) FSD and (d) PSD) phosphate adsorption (10 000× magnification).

The surface architecture of FSD and PSD before and after phosphate adsorption was analysed under the scanning electron microscope (10k× magnification) (Fig. 2). The SEM micrographs showed irregular structures of the particles and lamellar stratified surface of the shell dust that could provide a large surface area and plenty of active sites for phosphate adsorption. The shell dust had an additional spongy layer formation over the stratified surface, which indicates that surface precipitation has occurred during the adsorption process (Fig. 2b and d). The adsorption of phosphate on CaCO_3_-rich compounds is favoured in lower phosphate concentrations, and surface precipitation is favoured for high phosphate concentrations.^49,50^ The higher phosphate concentration (100 mg L^−1^) in the current instance resulted in surface precipitation and complexation, as observed in SEM micrographs. However, phosphate adsorption is expected to dominate at the start of the batch adsorption experiment.^49,50^ The surface precipitation of phosphate onto CaCO_3_-rich FSD and PSD produced Ca–P compounds, which were further confirmed through FTIR and XRD studies.

**Fig. 3:**
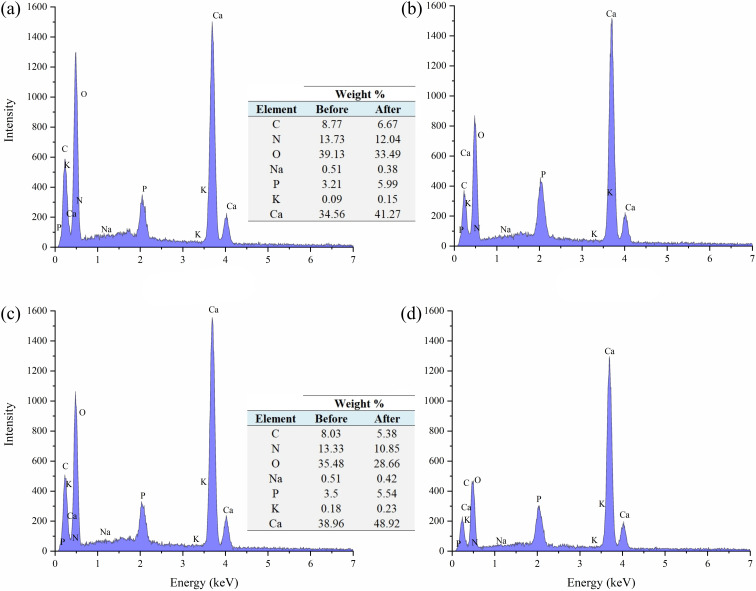
Energy-dispersive X-ray spectroscopy (EDS) images and elemental composition (% weight) of shell dust particles before ((a) FSD and (c) PSD) and after ((b) FSD and (d) PSD) phosphate adsorption.

**Fig. 4:**
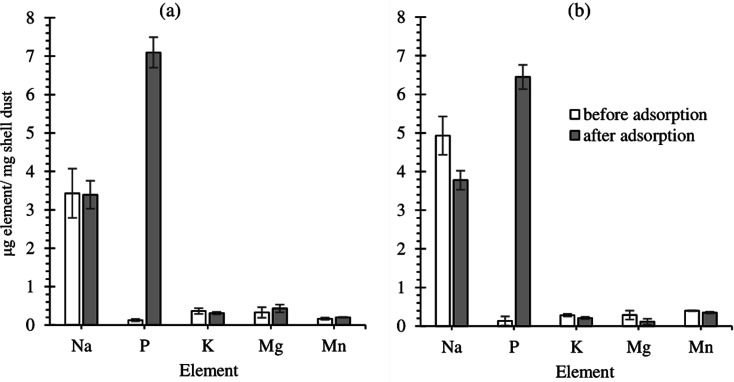
Elemental content of shell dust particles ((a) FSD and (b) PSD) before and after phosphate adsorption.

The elemental composition of FSD and PSD before and after the phosphate adsorption was estimated using EDS analysis. The prominent peaks in the EDS spectra correspond to C, O, P, K, N, and Na and significantly larger peaks for Ca in the untreated shell dust (Fig. 3). The elemental composition suggested that calcium, oxygen, nitrogen, and carbon were the major elements (by weight%) of the shell dust. The elemental profile of the phosphate-loaded shell dust on EDS showed differences in the elemental composition and increase of phosphate (1.86% for FSD and 2.04% for PSD by weight) that strongly suggests the adsorption of the phosphate ions onto the shell dust (Fig. 3). The result of the EDS analysis was also supported by element content estimation through ICP-OES, which indicated a significant increase in total phosphate content (μg P per mg of shell dust) following the adsorption experiment (Fig. 4).

**Fig. 5:**
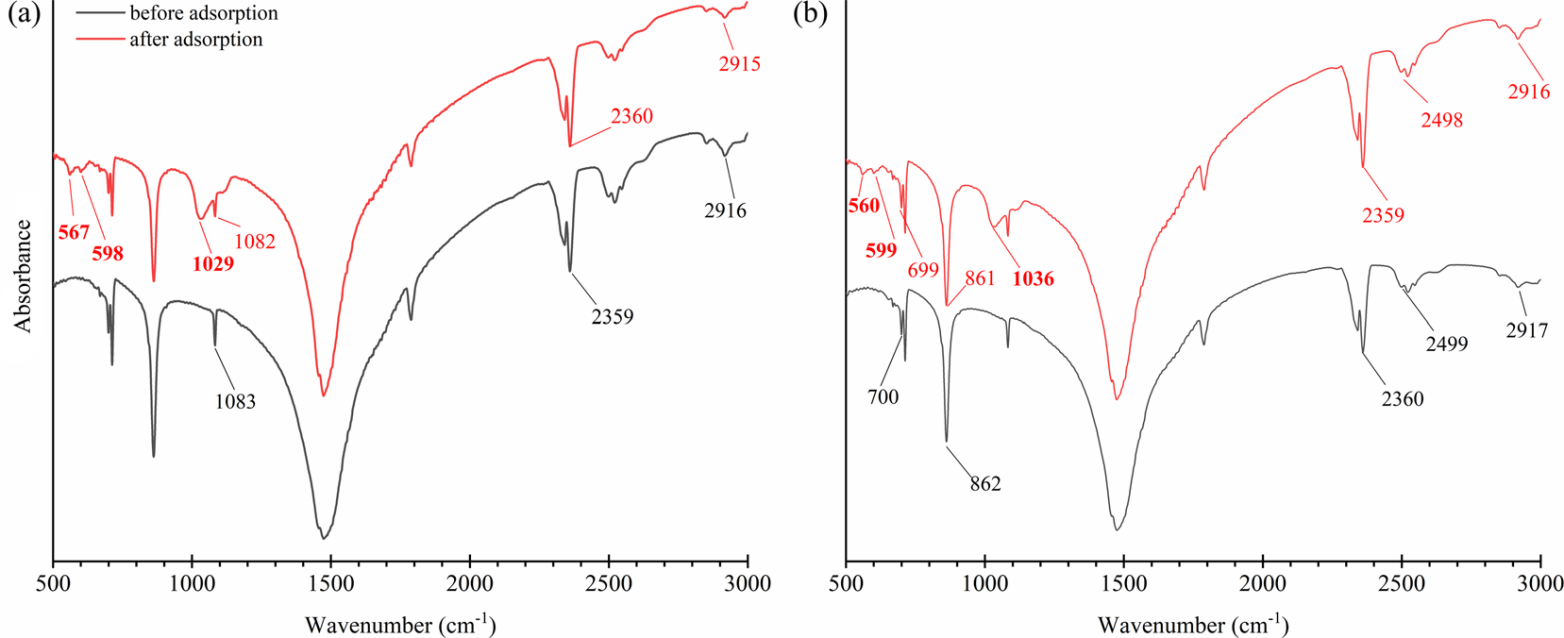
FTIR spectral analysis of shell dust particles ((a) FSD and (b) PSD) before and after phosphate adsorption. The appearance of additional absorbance bands after phosphate adsorption was indicated in bold, along with the change and shifts of absorbance bands compared with the FTIR spectra before phosphate adsorption. No absorbance scale is given in plots as the FTIR spectra were normalised and shifted parallel to the *x*-axis.

The FTIR spectral analysis (Fig. 5) indicated the presence of C–O bending in CO_3_^2−^ at ∼712 cm^−1^, *v*_2_ – out of plane vibration of CO_3_^2−^ at ∼860 cm^−1^, *v*_1_ – symmetric stretching of CO_3_^2−^ at 1082–1083 cm^−1^, and *v*_3_ – asymmetric stretching of CO_3_^2−^ at 1475 cm^−1^, which are the characteristics of CaCO_3_ (ref. 51) present on the shell dust. Besides, *cis*-C–H out-of-plane bend at ∼700 cm^−1^, asymmetric stretching mode of vibrations of O–H bonds at ∼2360 cm^−1^, and methylene C–H stretch at 2915–2918 cm^−1^ were also observed.^52–54^ The FTIR spectrum of phosphate adsorbed FSD and PSD showed addition and changes in the wavenumber and/or absorbance of characteristics bands, which indicate the addition and/or replacement of functional groups of the shell dust during the phosphate adsorption process.^10,49^ Additionally, the PO^3−^_4_ bands at 560–600 cm^−1^ and ∼1030 cm^−1^ (antisymmetric stretching)^42,55,56^ were appeared following the adsorption experiment, indicating phosphate adsorption onto FSD and PSD.

**Fig. 6:**
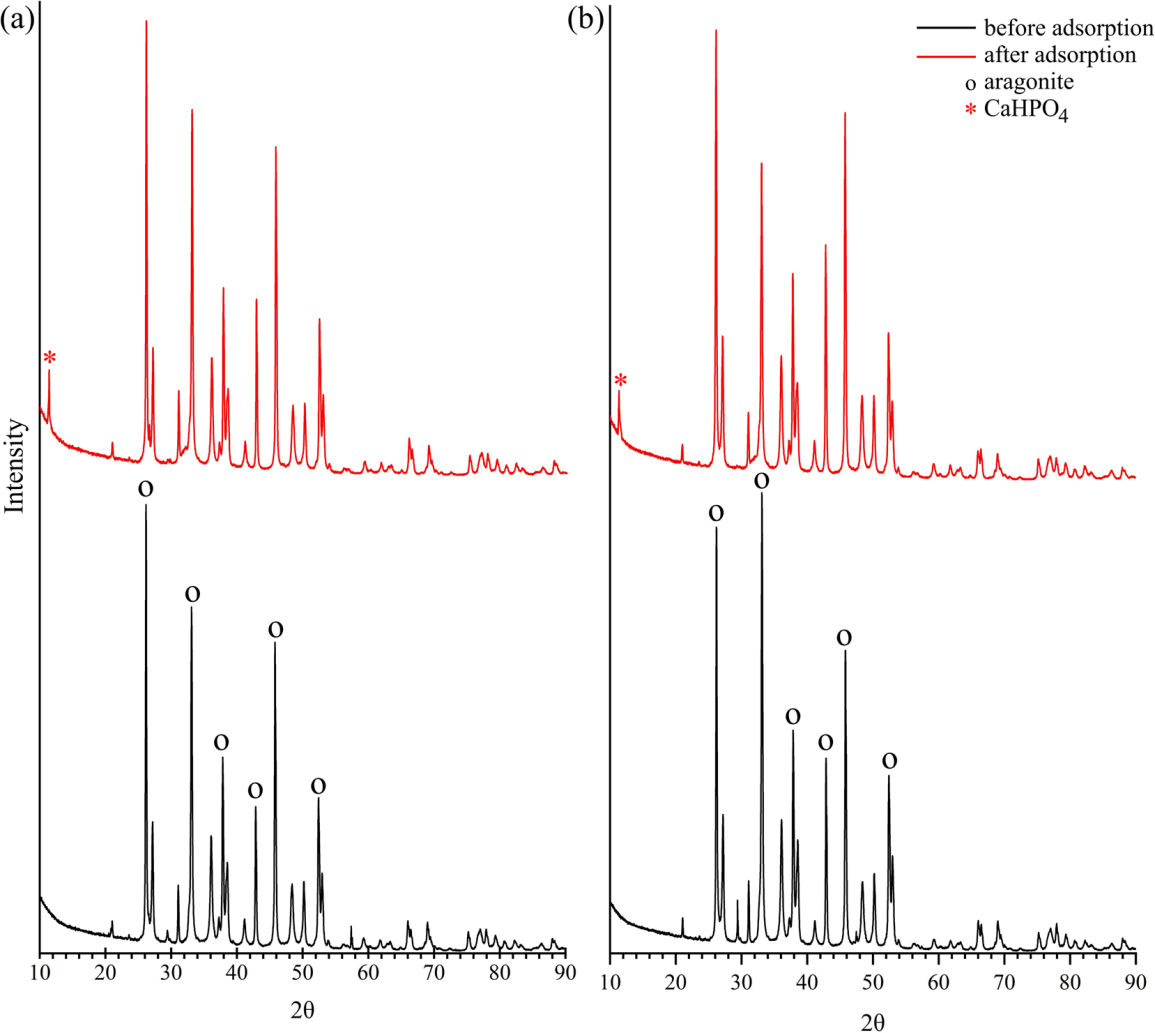
X-ray diffraction (XRD) patterns of the shell dust particles ((a) FSD and (b) PSD) before and after phosphate adsorption.

The X-ray diffractogram of the FSD and PSD indicated the presence of the aragonite phase of CaCO_3_ crystals (Fig. 6), which matched with an earlier observation during the characterisation of *F. bengalensis* and *P. globosa* shell dust.^40^ Following the batch adsorption experiment, an additional peak appeared near approximately 10 2*θ* degrees (Fig. 6), which was indicative of the appearance of dicalcium phosphate (CaHPO_4_) in the shell dust.^42,57^ A similar XRD peak was observed in the earlier studies, where phosphate was adsorbed onto CaCO_3_-rich carbonation cake.^42^

### 3.2. Effect of biosorbent dose

**Fig. 7:**
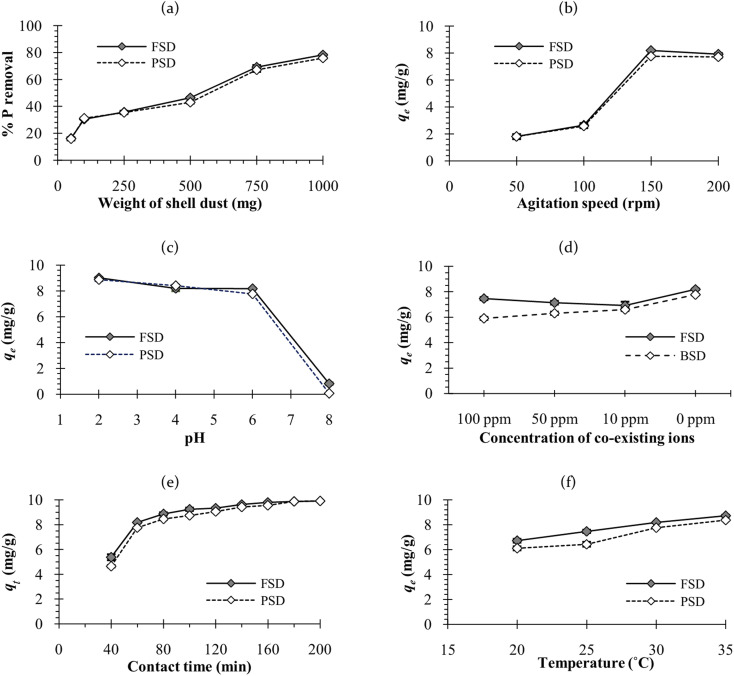
Effect of different variables in the phosphate ion removal efficiency of FSD and PSD – (a) weight of shell dust [fixed parameters: 30 °C, 150 rpm, 60 minutes, 100 mL phosphate solution of 100 mg L^−1^ concentration], (b) agitation speed [fixed parameters: 30 °C, 60 minutes, 1000 mg shell dust, 100 mL phosphate solution of 100 mg L^−1^ concentration], (c) pH [fixed parameters: 30 °C, 150 rpm, 60 minutes, 1000 mg shell dust, 100 mL phosphate solution of 100 mg L^−1^ concentration], (d) presence of other ions (NO_2_^−^, NO_3_^−^ and NH_4_^+^) under different concentrations [concentration of phosphate ion (100 mg L^−1^) remained same in all instances], (e) contact time [fixed parameters: 150 rpm, 1000 mg shell dust, 100 mL phosphate solution of 100 mg L^−1^ concentration] and (f) temperature [fixed parameters: 150 rpm, 60 minutes, 1000 mg shell dust, 100 mL phosphate solution of 100 mg L^−1^ concentration].

The amount of shell dust in the batch adsorption experiment has affected the phosphate removal percentage. As shown in Fig. 7a, percent phosphate removal increased with elevating amount of shell dust from 50 mg to 1000 mg (from 16.2 ± 0.9 to 78.3 ± 0.5% for FSD and 15.9 ± 1 to 75.9 ± 0.4% for PSD). The improved phosphate removal percentage can be attributed to the increased total surface area, and more adsorption sites.^23^ An earlier study on the *F. bengalensis* and *P. globosa* shell dust characterisation^40^ estimated an average mesopore size of 33.18 and 12.66 nm and pore volume of 0.079 and 0.043 cm^3^ g^−1^ for FSD and PSD, respectively. Additionally, the BET (Brunauer–Emmett–Teller) surface areas of FSD and PSD were estimated as 10.14 and 8.43 m^2^ g^−1^, respectively.^40^ The apparently larger mesopore size, pore volume, and surface area might result in comparatively higher phosphate removal efficiency of FSD.

### 3.3. Effect of agitation speed

The adsorption capacity of the shell dust increased with the agitation speed of 50 to 150 rpm (from 1.8 ± 0.2 to 8. 2 ± 0.1 mg g^−1^ for FSD and from 1.8 ± 0.2 to 7.81 ± 0.1 mg g^−1^ for PSD); however, the adsorption capacity remained roughly constant with further increase of the agitation speed (Fig. 7b). The escalation in the phosphate adsorption can be attributed to the external mass transfer that was amplified with increasing the agitation speed, resulting in the increase of solute transport from the solution to the adsorbent sites^58^ of the shell dust. Similar results were observed during the phosphate adsorption onto MIEX resin, where further increase in phosphate removal efficiency was negligible above the agitation speed of 150 rpm.^58^

### 3.4. Effect of pH

The affinity of phosphate ions toward the binding surface of biosorbents differs with the pH of the solution.^23^ In the current instance, phosphate removal was most favoured in the pH range of 2–6, and the maximum phosphate adsorption capacity was observed at pH 2 (9 ± 0.1 mg g^−1^ for FSD and 8.9 ± 0.1 mg g^−1^ for PSD) (Fig. 7c). At pH 8, phosphate removal by FSD and PSD decreased by almost 80% than at pH 2. The differences in the phosphate removal efficiency of FSD and PSD at different pH were related to the dissolution of Ca^2+^ ions from the shell dust and the polyprotic nature of phosphate.^59^ The lower phosphate removal in the higher pH can occur for the weak affinity of phosphate ions toward the adsorption sites of shell dust induced by intense competition with OH^−^ ions in the higher pH of the phosphate solution.^60^ The negatively charged sites dominate in the higher pH, which enhanced the repulsion effect that decreased the amount of phosphate adsorption, along with the decrease of Ca^2+^ with increasing initial pH.^59^ On the other side, higher adsorption of phosphate ions onto the shell dust at lower pH can be described by the prevalence of HPO^−^_4_, H_2_PO^−^_4_, and PO^3−^_4_ in the acidic medium.^23^ Additionally, the positively charged surface sites formed due to lower initial pH on the adsorbent favour the phosphate adsorption due to the electrostatic attraction,^59^ and calcium phosphate precipitates can be formed in the amorphous form at lower pH.^42,59^ The higher adsorption with the decreasing pH can be explained by the reactions of Ca^2+^ with different phosphate species (Ca^2+^ + H_2_PO^−^_4_ → CaH_2_PO^+^_4_; Ca^2+^ + HPO^2−^_4_ → CaHPO_4_ and Ca^2+^ + PO^3−^_4_ → CaPO^−^_4_).^42^ The formation of calcium phosphates was also established by the results of FTIR and XRD studies (Fig. 5 and 6).

### 3.5. Effect of coexisting ions

Anions like nitrite, nitrate, and cations like ammonium ions often coexist with phosphate in the wastewater, which may compete with the phosphate ion for the adsorption sites^10^ of the shell dust. Therefore, the effect of these coexisting ions on the phosphate adsorption capacity of FSD and PSD was studied using different concentrations of coexisting ions. The adsorption capacity (*q*_e_) of the shell dust was reduced in the high coexisting ion concentration as compared with the adsorption capacity in the absence of coexisting ions (from 8.2 ± 0.1 to 7.5 ± 0.1 mg g^−1^ in the case of FSD and 7.8 ± 0.03 to 5.9 ± 0.1 mg g^−1^ in case of PSD) at pH 6 (Fig. 7d). Along with phosphate, nitrogen is another key element responsible for eutrophication. As FSD and PSD efficiently removed phosphate even in the presence of nitrogen-containing ions, the prospective biosorbents can be applied to remove phosphate from industrial effluents as well as highly eutrophicated water bodies. The earlier studies showed a similar result, where the presence of nitrate ions did not alter the phosphate adsorption onto cerium/manganese oxide-based nanocomposites,^46^ cerium oxide nanoparticle functionalised lignin,^48^ basic oxygen furnace slag,^61^ dolomite and hydroxyapatite.^62^

### 3.6. Effect of initial concentration of phosphate solution and analysis of adsorption isotherms

**Fig. 8:**
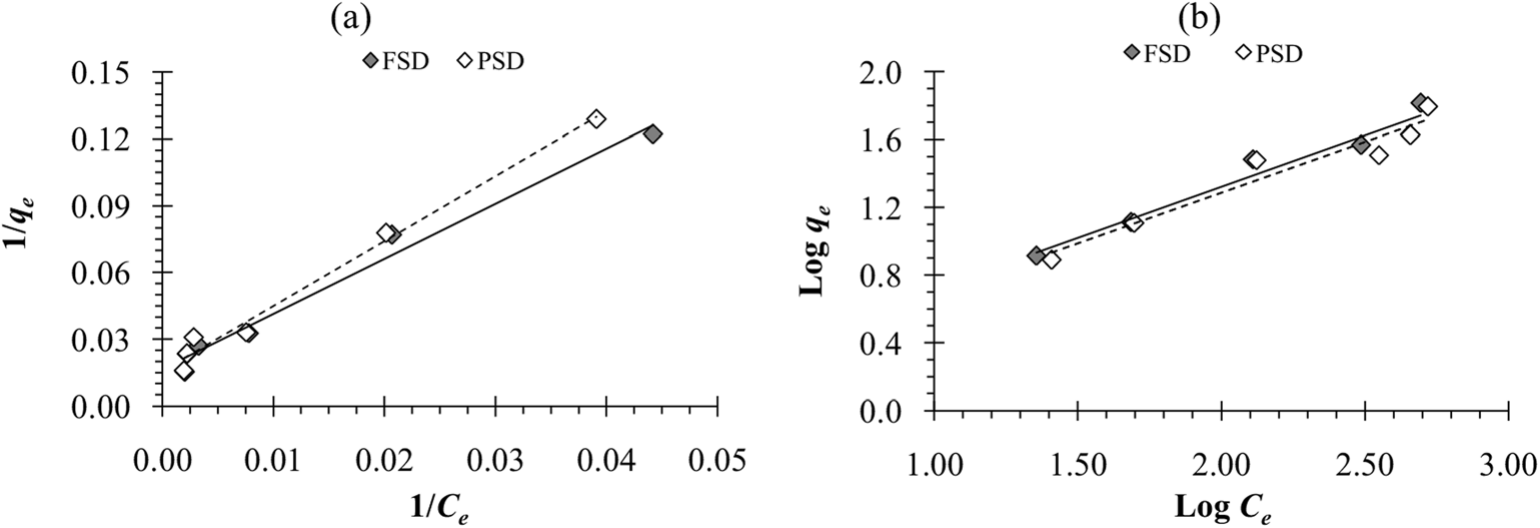
(a) Langmuir adsorption isotherm and (b) Freundlich adsorption isotherm of phosphate adsorption onto FSD and PSD at 30 °C.

The adsorption capacity of FSD and PSD was observed to be increased with a corresponding increase in initial phosphate ion concentration. Different phosphate ion concentrations (between 100 and 1000 mg L^−1^) were used in the experiment. The lowest adsorption was observed at the initial concentration of 100 mg L^−1^, while the highest adsorption was observed at 1000 mg L^−1^ concentration. However, the percent phosphate removal was decreased with the increasing initial phosphate concentration. The lower removal percentage can be attributed to the increased ratio of moles of phosphate against the available surface area of shell dust with increasing initial phosphate concentrations.^63^ As the dose of biosorbent was fixed in the current instance, the available phosphate adsorption sites remained constant on the shell dust, which decreased the percent phosphate removal with increasing initial phosphate concentration.^63^ Similar results were reported for the phosphate adsorption onto iron hydroxide-eggshell.^63^ The adsorption isotherms are essential for the adsorption studies as the isotherms express the relation between adsorbate concentration and their accumulation onto the adsorbent surface and estimate the biosorption capacity of the adsorbent. The linear forms of Langmuir and Freundlich isotherm were used to fit the obtained experimental data to evaluate the performance of FSD and PSD at 30 °C, and the plots are shown in Fig. 8. Adsorption isotherm constants for phosphate adsorption onto snail shell dust were derived from the isotherm plots and presented in Table 2. In terms of the correlation coefficient values, the Langmuir isotherm is the best-fitted model for the experimental data. The conformity of phosphate adsorption data to Langmuir isotherm can be explained as a homogenous adsorption process that led to the monolayer binding of phosphate ions^10^ onto the surface of shell dust. The maximum adsorption capacity (*q*_max_) calculated by the function was 62.50 mg g^−1^ and 66.66 mg g^−1^ for FSD and PSD, respectively. A comparative table (Table 3) is provided for the adsorption capacities of different recently used biomaterials to remove phosphate from wastewaters. The values of the equilibrium parameter, *R*_L_, were in the range of 0 < *R*_L_ < 1 (0.13–0.62 for FSD and 0.15–0.66 for PSD), indicating that FSD and PSD were favourable biosorbents to remove phosphate ions from aqueous solution.^10^

**Table 2:** Adsorption isotherm constants for phosphate adsorption onto FSD and PSD

SSD	Langmuir isotherm					Freundlich isotherm				
	Intercept	Slope	*q*_max_ (mg g^−1^)	*K*_L_	*R* ^2^	Intercept	Slope	1/*n*	*K*_F_	*R* ^2^
FSD	0.016	2.476	62.50	0.006	0.982	0.114	0.603	0.603	1.300	0.956
PSD	0.015	2.913	66.66	0.005	0.988	0.088	0.599	0.599	1.225	0.936

**Table 3:** Reports on the removal/adsorption of phosphate ions from synthetic or industrial wastewater by different biomaterials

S. no.	Biomaterial	P removal (*q*_max_ mg g^−1^)
1	Sugarcane bagasse derived cellulose^26^	21.4
2	Chitosan-calcite biosorbent^64^	21.5
3	Chitosan hydrogel beads^65^	30.1
4	Zirconium loaded okara^23^	44.13
5	Magnesium oxide nanoflake-modified diatomite^66^	52.08
6	Wheat straw^24^	46
7	Cotton stalk^25^	52
8	Hydrotalcite^67^	60
9	Modified chitosan beads^10^	60.6
10	Giant reed^68^	61
11	Wheat stalk^25^	61
12	**Dust of *F. bengalensis* shell (FSD)** ^**current study**^	**62.50**
13	**Dust of *P. globosa* shell (PSD)** ^**current study**^	**66.66**
14	Chemically modified sawdust^44^	82.60
15	Amine cross-linked tea wastes^69^	98.72

### 3.7. Effect of contact time and analysis of kinetic models

**Fig. 9:**
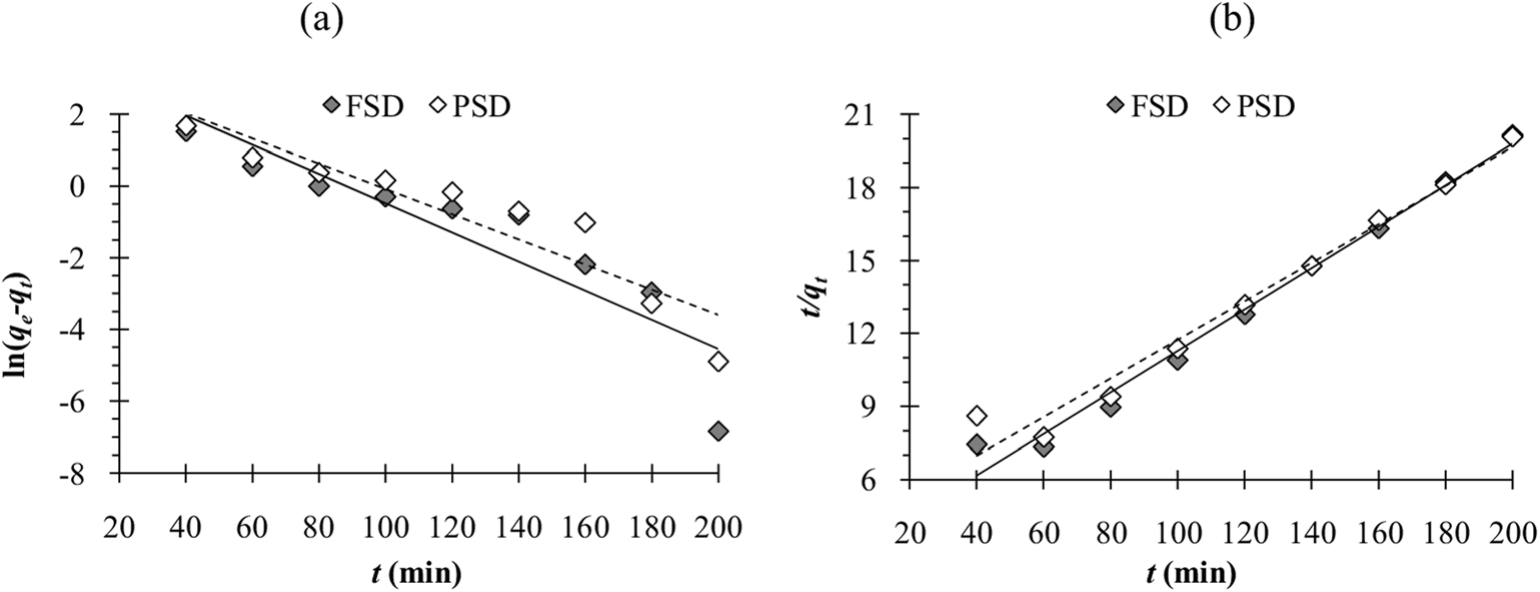
(a) Pseudo-first order and (b) pseudo-second order adsorption kinetics obtained from the experimental data.

The results of phosphate adsorption onto FSD and PSD showed rapid uptake of phosphate ions for the first 80 minutes, and phosphate uptake was reduced as the equilibrium time approached following this time (Fig. 7e). The more or less constant phosphate adsorption efficiency of FSD and PSD after 80 minutes of contact time can be attributed to the decrease in the number of vacant sites on the shell dust surface. Additionally, remaining vacant adsorption sites could be difficult to occupy by the phosphate ions due to repulsive forces between the phosphate ions on the solution and shell dust.^63^ The initial adsorption is rapid, generally when the surface reaction mediates the adsorption process, and the adsorption decreases with the increasing time due to lower active adsorption time. The process is consistent with previous studies, where iron hydroxide-eggshells,^63^ zirconium loaded okara,^23^ and chitosan hydrogel beads^65^ were used as adsorbents of phosphate ions. The linear form pseudo-first-order and pseudo-second-order kinetic models were analysed based on the experimental data, which is necessary to identify the type of adsorption mechanism in the experimental system. The linear form of pseudo-first order and pseudo-second order kinetics models of the phosphate adsorption are presented in Fig. 9. The parameters of both kinetic models were calculated using the intercepts and slopes of Fig. 7 and are shown in Table 4. The experimental data were fitted better by the pseudo-second order model (*R*^2^ – 0.985 for FSD and 0.972 for PSD) than the pseudo-first order model (*R*^2^ – 0.812 for FSD and 0.861 for PSD). The high *R*^2^ values of the pseudo-second order model suggested that the chemical adsorption process might occur between the shell dust and phosphate ion.

**Table 4:** Pseudo-first order (PFO) and pseudo-second order (PSO) adsorption kinetic parameters of phosphate ion adsorption onto FSD and PSD

	PFO					PSO				
	Intercept	Slope	*q*_e_ (mg g^−1^)	*k*_1_	*R* ^2^	Intercept	Slope	*q*_e_ (mg g^−1^)	*k*_2_	*R* ^2^
FSD	3.588	−0.04	36.16	−0.0002	0.812	2.771	0.085	11.76	0.0026	0.985
PSD	3.429	−0.035	30.85	−0.0001	0.861	3.824	0.079	12.66	0.0016	0.972

### 3.8. Effect of temperature and analysis of thermodynamics parameters

**Fig. 10:**
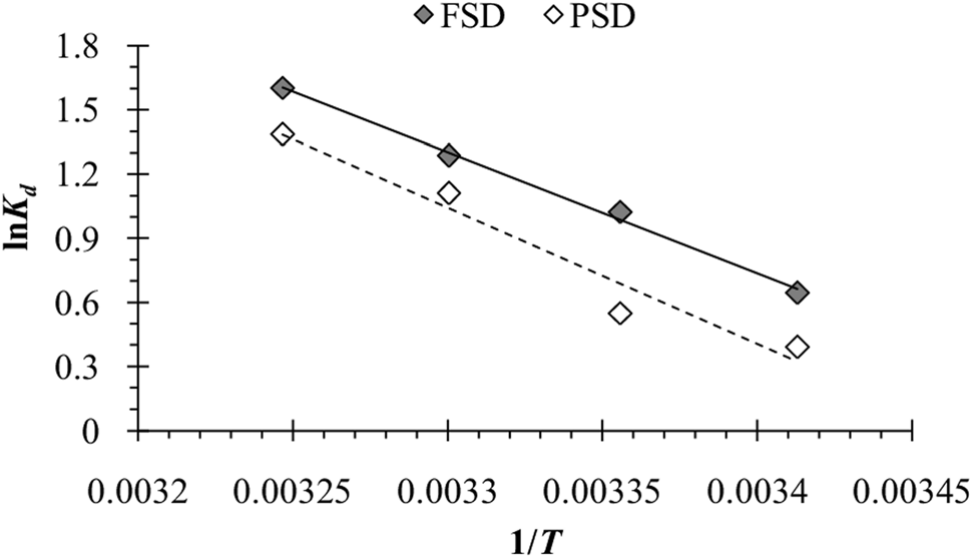
A plot of ln *K*_d_ against 1/*T* for phosphate adsorption onto FSD and PSD.

The temperature of the batch adsorption experiment had a significant effect on the phosphate adsorption onto FSD and PSD. The phosphate adsorption was monitored at four temperatures, and maximum adsorption capacity was observed at 35 °C, which was increased by more than 17.5% for FSD and 20% for PSD than that of 20 °C (Fig. 7f). As the adsorption of phosphate onto FSD and PSD was chemical in nature, the growing temperature of the solution may increase the solubility of shell dust which provides more calcium complexes for phosphate ion precipitation.^63^ A similar trend of phosphate removal was observed when iron hydroxide-eggshell,^63^ modified chitosan beads,^10^ and zirconium loaded okara^23^ were used as an adsorbent of phosphate ions from wastewater. The thermodynamic parameters – Δ*G*° (Gibbs free energy, J mol^−1^), Δ*H*° (enthalpy of the system, J mol^−1^), and Δ*S*° (entropy, J mol^−1^ K^−1^) of the phosphate adsorption process were analysed using the experimental data and appropriate equations (Table 1). Δ*H*° and Δ*S*° were calculated from the slope and intercept of the plot of ln *K*_d_*versus* 1/*T* (Fig. 10), which showed linearity with high correlation coefficient values (*R*^2^ for FSD – 0.996, and *R*^2^ for PSD – 0.956). Table 5 represents the values of thermodynamic parameters – Δ*G*° at different temperatures, Δ*H*° and Δ*S*°. The negative values of Δ*G*° indicated that the phosphate adsorption onto FSD and PSD was spontaneous and feasible in nature. The values of Δ*G*° increased with decreasing temperature (Table 5), suggesting the presence of more absorbable phosphate ions with increasing temperature. The values of Δ*H*° and Δ*S*° were positive in the adsorption of phosphate onto the shell dust, indicating the endothermic nature of the adsorption process, and randomness was increased at the solid/liquid interface during the adsorption process, which reflected good affinity of FSD and PSD toward phosphate ion.

**Table 5:** Thermodynamics parameters of phosphate adsorption onto shell dust

Shell dust	Temp. (K)	*K*_L_	Δ*G*° (kJ mol^−1^)	Δ*H*° (kJ mol^−1^)	Δ*S*° (kJ mol^−1^)	*R* ^2^
FSD	293	1.905	−1.570	47.08	166.2	0.996
	298	2.783	−2.535			
	303	3.616	−3.238			
	308	4.965	−4.104			
PSD	293	1.477	−0.950	53.16	184.07	0.956
	298	1.731	−1.359			
	303	3.036	−2.797			
	308	3.995	−3.547			

The wastewater originating from the municipal sewage and aquaculture farms are featured by high phosphate content.^1,70^ In case of eutrophicated pond, lake and allied wetlands, the load of phosphate is a crucial factor which deserve appropriate management.^1,11,70^ Removal of phosphate from eutrophicated water using shell dust may provide a feasible option too. Besides, the CaCO_3_ would supplement the liming process that may stabilize suspended matters as well as promote autotrophic growth.^71^ Thus, the snail shell dust will provide dual benefits of purification of the water as well as the removal of phosphate from the water, with prospective uses elsewhere. For instance, the fertiliser industry utilises more than 80% of mined phosphate rocks around the globe, which is an essential part of modern agriculture.^72^ Due to excessive mining of phosphate rocks, the global phosphate reserve is forecasted to be exhausted within the next 100 years.^73^ The conventional methods of phosphate recovery from wastewaters produce phosphate-containing sludge,^74^ and the sludge usually has a low phosphate concentration to be considered as a phosphate fertiliser.^75^ Although the chemical methods are rapid in capturing phosphate ions from water by adding aluminium, calcium, and ferric chemicals to water,^76–78^ the aluminium and ferric chemicals are not appropriate as fertilisers as the bonding of phosphate to such chemicals is too strong to be absorbed by plants.^79^ Previous studies have shown the utilisation of calcium carbonate to improve sandy soil,^80^ increase the pH of acidic soils with low fertility, and as an amendment to facilitate forest restoration.^81^ The shells of edible snails, *F. bengalensis* and *P. globosa*, consist of more than 87% calcium carbonate^40^ that can be used as a liming source in such instances. As the phosphate-loaded adsorbents slowly release phosphorus, they are suitable for providing phosphorus to the soil. For example, a recent study experimentally showed that the application of phosphate-loaded CaCO_3_ composite as fertiliser had increased the growth and yield of vegetables in sandy soils.^79^ Therefore, the use of phosphate adsorbed FSD and PSD (with high phosphate content) as a fertiliser and liming agent can be considered as an alternative strategy for phosphate recycling and improvement of soil property. However, the presence of contaminants like heavy metals, dyes and organic content may interfere with the phosphate removal process. Since the shell dust is also an effective heavy metal adsorbent,^22,36–39^ the synergistic or simultaneous removal of phosphate and metal may also be possible. Therefore, further studies using the snail shell dust can be carried out to justify its efficacy as adsorbent of multiple pollutants.

## 4. Conclusion

The effects of various parameters on the phosphate adsorption were systematically examined, and the experimental data fitted well with the Langmuir adsorption isotherm model, indicating monolayer adsorption with the maximum adsorption capacity of 62.50 and 66.66 mg phosphate per gram of FSD and PSD, respectively. The pseudo-second order kinetic model was well fitted, indicating the chemical adsorption process. The thermodynamic parameters indicated that the adsorption mechanism of phosphate onto shell dust was spontaneous, feasible, and endothermic. Additionally, the SEM, EDS, ICP-OES, FTIR, and XRD studies confirmed the adsorption of phosphate onto the FSD and PSD. Hence, the experimental results imply that shell dust derived from the waste shells of edible freshwater snails could potentially be employed as a biosorbent of phosphate ions from wastewater, industrial effluents, and eutrophicated water bodies. On the other hand, the phosphate-loaded FSD and PSD can be utilised as fertiliser and liming agent to improve the soil property. With the increasing importance of “green thinking” and the “waste made useful” paradigm, the availability and low cost of waste shells and their use in wastewater treatment may provide a viable option in terms of sustainability from industrial, ecological, and economic perspectives.

## Data availability

The data of this experiment and the results of this article can be made available upon authentic and reasonable request.

## Author contributions

Procurement of samples, conceptualisation, preliminary and final draft – GA; procurement of samples, execution of the experiment, data analysis and draft – PP; experiment – SP.

## Conflicts of interest

As authors of this article, we declare that we do not have any conflict of interest.

## Supplementary Material
